# Correlation of Prostatic Urethral Angle with the Severity of Urinary Symptom and Peak Flow Rate in Men with Small Prostate Volume

**DOI:** 10.1371/journal.pone.0104395

**Published:** 2014-08-15

**Authors:** Dong Hyuk Kang, Joo Yong Lee, Yoon Soo Hah, Doo Yong Chung, Dae Hun Lee, Kang Su Cho

**Affiliations:** 1 Department of Urology, Yangpyeong Health Center, Yangpyeong, Korea; 2 Department of Urology, Severance Hospital, Urological Science Institute, Yonsei University College of Medicine, Seoul, Korea; 3 Department of Urology, Severance Check-up, Yonsei University Health System, Seoul, Korea; 4 Department of Urology, Gangnam Severance Hospital, Urological Science Institute, Yonsei University College of Medicine, Seoul, Korea; H. Lee Moffitt Cancer Center & Research Institute, United States of America

## Abstract

**Purpose:**

To evaluate the effects of prostatic anatomical factors on male lower urinary tract symptoms (LUTS) and the peak flow rate (Qmax) in patients with small prostate volume (PV).

**Materials and Methods:**

Records were obtained from a prospectively maintained database of first-visit men with LUTS. Patients whose total PV (TPV) was greater than 30 mL were excluded; 444 patients were enrolled in the study. The TPV, transitional zone volume (TZV), transitional zone index (TZI), intravesical prostatic protrusion (IPP), and prostatic urethral angle (PUA) were measured by transrectal ultrasonography. LUTS were evaluated using the International Prostate Symptom Score (IPSS) and the Overactive Bladder Symptom Score (OABSS) questionnaires. Uroflowmetric measurements were also made.

**Results:**

PUA (r = 0.269, P<0.001), TZV (r = 0.160, P<0.001), and TZI (r = 0.109, P = 0.022) significantly correlated with the IPSS. Qmax (r = −0.334, P<0.001) and OABSS (r = 0.211, P<0.001) correlated only with PUA. In a multivariate regression analysis, PUA and age were independently associated with IPSS, OABSS, and Qmax. For IPSS of 20 or greater, the area under the ROC curve (AUC) of PUA was 0.667 and the cut-off value was 43.7°. When Qmax was 10 mL/s or less, the AUC of PUA was 0.664 and the cut-off value was 43.5°.

**Conclusions:**

PUA has a significant association with symptom severity and Qmax among prostatic anatomical factors analyzed in men with LUTS and small PV. PUA should be considered as an important clinical factor in male LUTS management. Furthermore, the impact of PUA on response to medical treatment and disease progression needs to be investigated.

## Introduction

Benign prostatic hyperplasia (BPH) that includes benign prostatic enlargement (BPE) and benign prostatic obstruction (BPO) has conventionally been considered a major factor in male lower urinary tract symptoms (LUTS) [1]. However, the pathophysiology of male LUTS is highly complex and multifactorial, and recently the importance of other causal factors including changes in the bladder and prostate as well as in related structures such as the pelvic vasculature and innervation has been highlighted [2]. To reflect this viewpoint, the European Association of Urology (EAU) replaced ‘LUTS suggestive of BPH (LUTS/BPH)’ with ‘Non-neurogenic male LUTS including BPO’ in the latest version of the EAU guidelines for male LUTS [3].

Some reports demonstrate that the correlation between the absolute prostate volume (PV) and LUTS severity is weak [4]–[6]. Several researchers have instead emphasized the positive relationship between male LUTS severity and other prostatic anatomical factors such as intravesical prostatic protrusion (IPP), transitional zone index (TZI), and prostatic urethral angulation (PUA) [7]–[10]. Meanwhile, physicians frequently encounter men with LUTS and a PV less than 25 mL; the significance of the PV has been neglected or regarded as uncertain in the consultation and management of these patients.

The correlation between the absolute PV and male LUTS severity is undoubtedly weaker in patients with small PV. However, the relationship between LUTS severity and other anatomical prostatic factors have not yet been investigated in the clinical setting. Herein, we evaluate the effects of prostatic anatomical factors including the total PV (TPV), transitional zone volume (TZV), TZI, IPP, and PUA on LUTS and the peak flow rate (Qmax) in patients with a PV of 30 mL or less.

## Materials and Methods

### 1. Patient cohort

**Figure 1:**
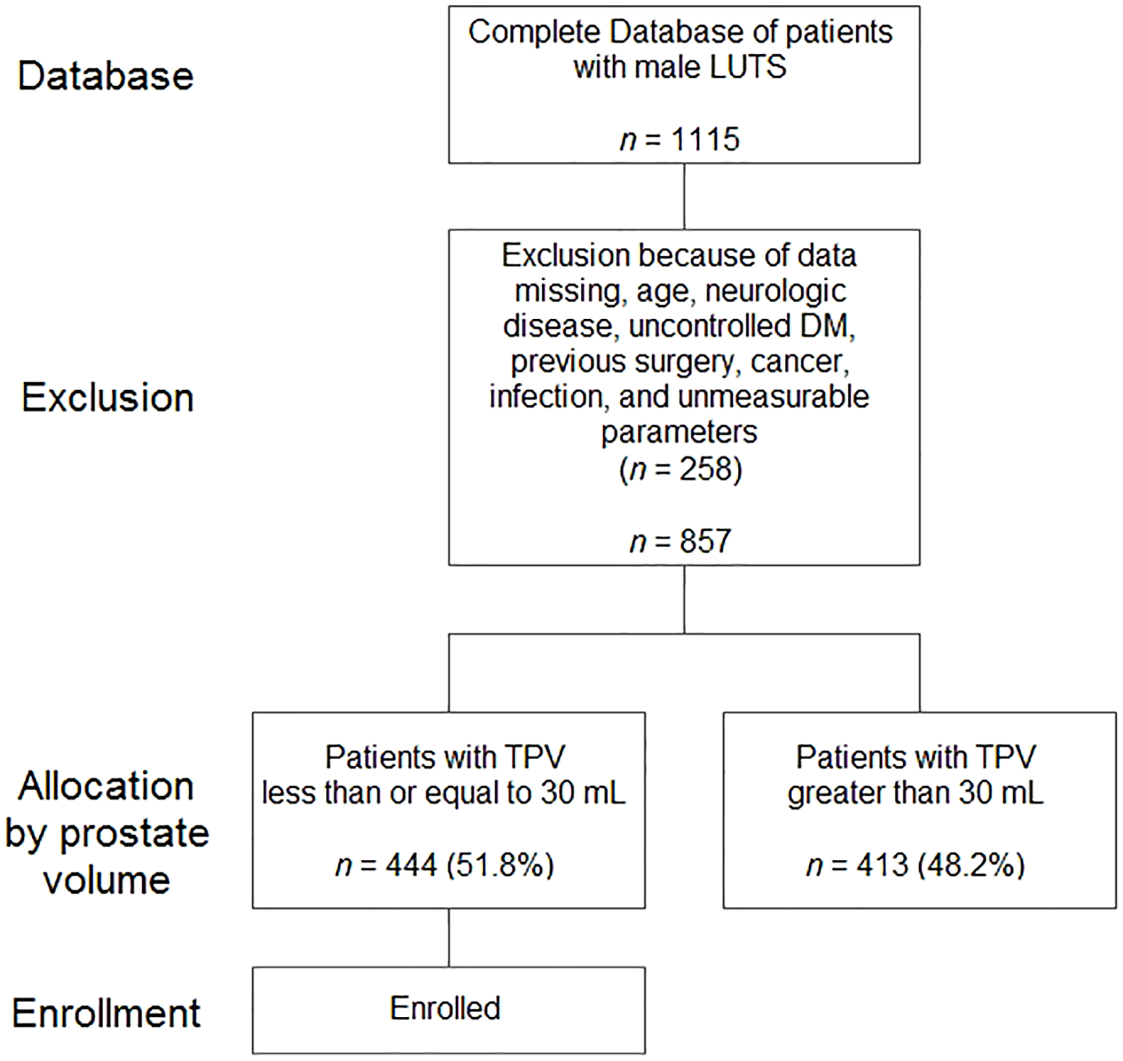
Flow diagram of study enrollment.

Medical records were obtained from a prospectively maintained database of first-visit male patients with LUTS/BPH between April 2010 and December 2012 at our outpatient clinics. During this period, 1115 patients were registered in our database. Inclusion criteria for subjects included (1) age from 40 to 80 years, (2) a TPV 30 mL or less, and (3) an interest and ability to participate in this study. Exclusion criteria included (1) bladder or prostate cancer, (2) uncontrolled diabetes mellitus, (3) neurologic disease that could influence voiding symptoms, (4) history of previous lower urinary tract surgery, (5) urogenital infections, or (6) unmeasurable PUA due to severe calcification or a large volume. In sum, 444 patients were eligible for analysis (Fig. 1). Written informed consent was given by all participants for their clinical records to be used in this study.

### 2. Good clinical practice protocols

The study was performed in agreement with applicable laws and regulations, good clinical practices, and ethical principles as described in the Declaration of Helsinki. This study was approved by the institutional review board at Severance Hospital prior to the study (Approval Number: 4-2013-0845).

### 3. Assessment of prostatic anatomical factors

**Figure 2:**
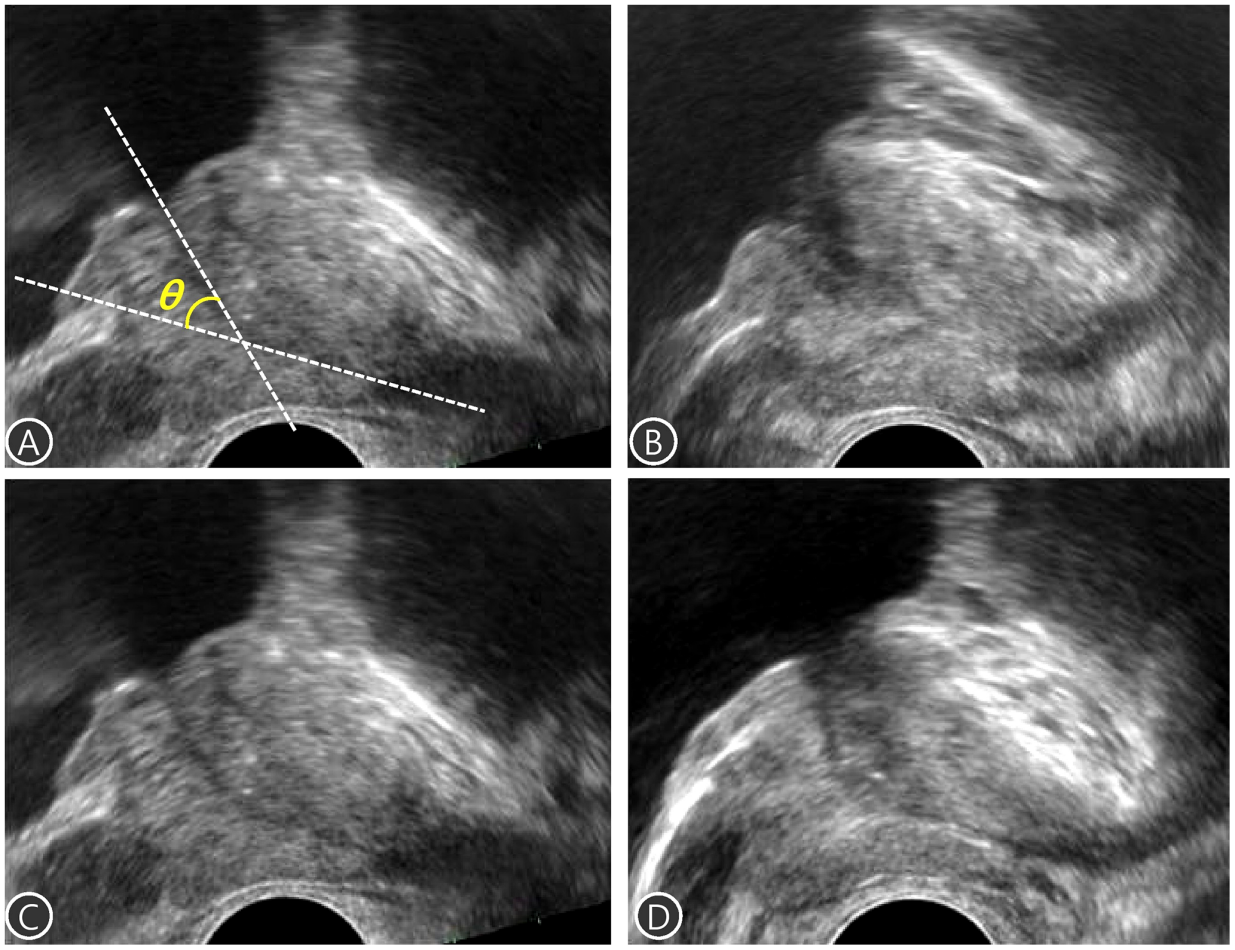
The measurements of prostatic urethral angulation (PUA). (A) The PUA was defined as the angle formed by 2 rays of both proximal and distal prostatic urethra on the midsagittal plane image, (B) to (D) The representative images showing individual differences in PUA. The measured value of PUA was 31° for (B), 44° for (C), and 58° for (D).

PV was assessed by a single urologist via transrectal ultrasonography (TRUS) using a single ultrasound machine (Prosound Alpha 5 SV, Hitachi Aloka, Tokyo, Japan). The TPV and TZV were measured using the prolate ellipsoid formula (height×width×length×π/6). Other prostatic anatomic factors including the IPP and TZI were also measured. Transition zone index was calculated according to the formula, TZI = TZV/TPV [10]. IPP is the vertical distance from the protruded tip to the circumference of the bladder at the prostate base taken at the mid-sagittal view [11]. The PUA was defined as the angle formed by 2 rays of both proximal and distal prostatic urethra on the midsagittal plane image, and which was taken with the posterior wall of the prostate positioned as flat as possible to minimize the influence of pressure from the rectal probe as shown in Fig. 2 [8], [12]. Two independent urologists (DHK and JYL) calculated the PUA, and the average of these two values was scored for greater accuracy.

### 4. Assessment of LUTS

LUTS were evaluated using the International Prostate Symptom Score (IPSS) and the Overactive Bladder Symptom Score (OABSS) questionnaires. Qmax and postvoid residual volume (PVR) measurements were also made. Uroflowmetric measurements were made with the patient in the standing position using Bluetooth uroflowmetry (Urodyn+; Mediwatch UK, Ltd., Ruby, United Kingdom); maximal flow measurements were discarded if the voided volume was less than 125 mL, and both uroflowmetric and PVR measurements were repeated. PVR was measured using a bladder scanner (BioSon-500; MCube Tech, Seoul, Korea).

### 5. Interobserver Variability

Interobserver reliability for assessments of PUA among the 2 examiners was determined by calculating intraclass correlation coefficients (ICCs) according to a 2-way random effects model using R (R ver. 3.0.2, R Foundation for Statistical Computing, Vienna, Austria; http://www.r-project.org). An ICC value less than 0.20 was considered poor, 0.21–0.40 fair, 0.41–0.60 moderate, 0.61–0.80 substantial, and 0.81–1.00 very good.

### 6. Statistical analysis

Pearson's product-moment correlation test was used to assess the relationships between variables. Multivariate linear regression analysis was performed to analyze the independent association of age and prostatic parameters with IPSS, OABSS, and Qmax. Total IPSS was classified into post-micturition, storage, and voiding symptom domains. These subscores were also analyzed separately. Optimal cut-off values for symptom severity were identified from the Receiver Operator Characteristic (ROC) curves using Youden methods. Data were analyzed in R (R ver. 3.0.2, R Foundation for Statistical Computing, Vienna, Austria; http://www.r-project.org) and its OptimalCutpoints package for optimal cut-off value.

## Results

### 1. Patient population and demographics

A total of 444 patients were enrolled in the study. The mean age was 59.44±10.87 years. The mean PV, TZV, and TZI were 23.02±4.13 mL, 9.67±2.84 mL, and 0.42±0.09, respectively. The mean PUA was 43.25±7.47° and IPP was 0.82±1.30 mm. Patient characteristics and clinical parameters are summarized in Table 1.

**Table 1:** Patient characteristics and clinical parameters.

Number of patients enrolled	444
Age (years)	59.44±10.87
IPSS	
Total	16.49±7.26
Post-micturition symptoms	2.54±1.56
Voiding symptoms	7.40±3.88
Storage symptoms	6.56±3.36
OABSS	4.46±3.15
Uroflowmetry	
Qmax (mL/s)	15.39±7.63
PVR (mL)	23.75±34.97
TRUS	
TPV (mL)	23.02±4.13
TZV (mL)	9.67±2.84
TZI	0.42±0.09
IPP (mm)	0.82±1.30
PUA (°)	43.25±7.47
Serum PSA (ng/mL)	1.28±1.51
Number of comorbidities	
HTN (%)	142 (32.0)
DM (%)	73 (16.4)

IPSS, International Prostate Symptom Score; OABSS, Overactive Bladder Symptom Score; Qmax, peak flow rate; PVR, postvoid residual volume; TPV, total prostate volume; TZV, transitional zone volume; TZI, transitional zone index; IPP, intravesical prostatic protrusion; PUA, prostatic urethral angulation; PSA, prostate-specific antigen; HTN, hypertension; DM, diabetes mellitus; CVA, cerebrovascular accident.

### 2. Agreement among examiners

The PUA results obtained by the 2 examiners considered collectively were in high agreement, with an overall reliability of estimates of 90.8% (95% confidence interval, 0.888–0.926).

### 3. Pearson's product-moment correlation

**Figure 3:**
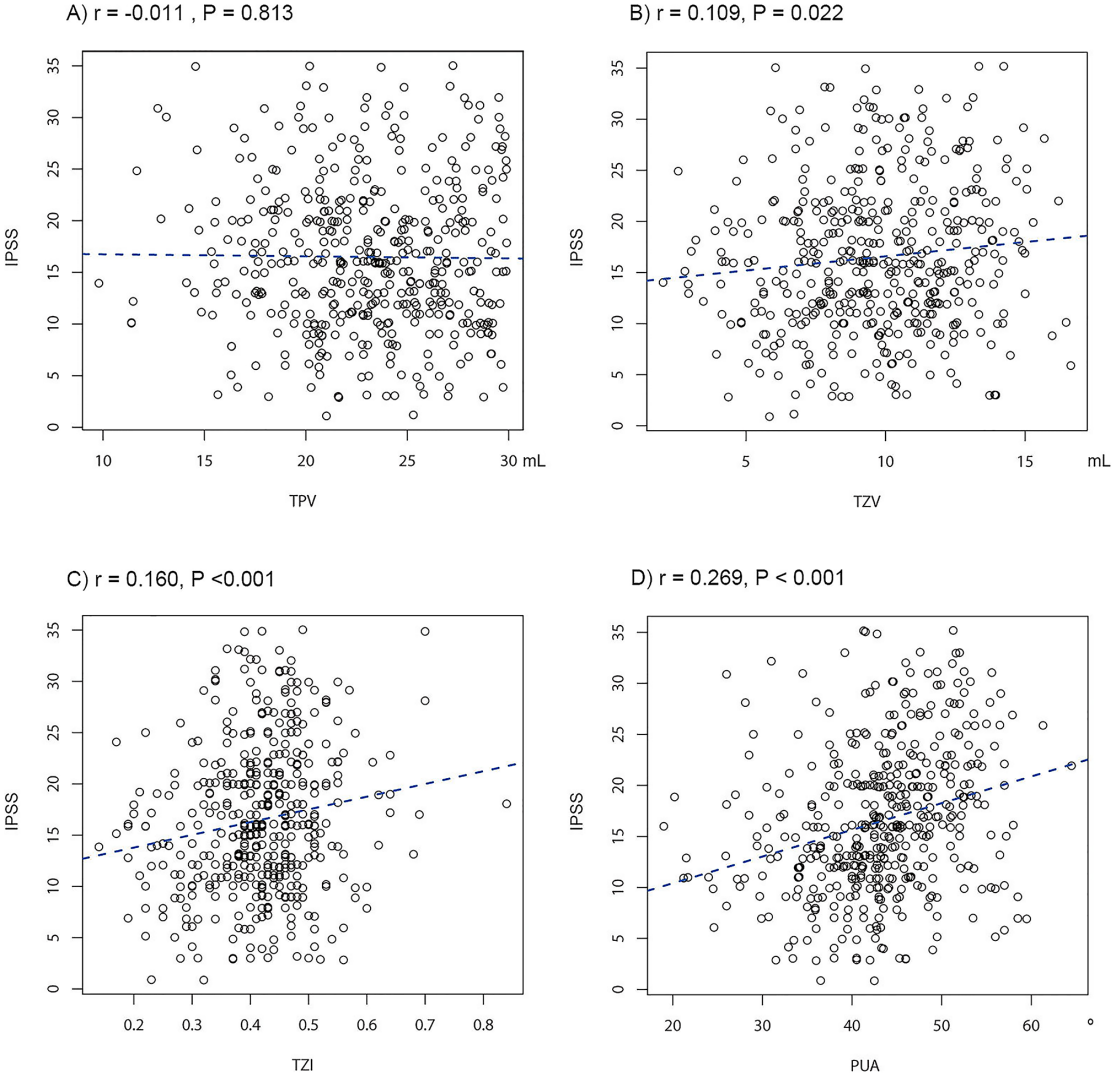
Correalation between prostatic anatomical factors and the International Prostate Symptom Score (IPSS). (A) total prostate volume (TPV), (B) transitional zone volume (TZV), (C) transitional zone index (TZI), and (D) prostatic urethral angulation (PUA).

**Figure 4:**
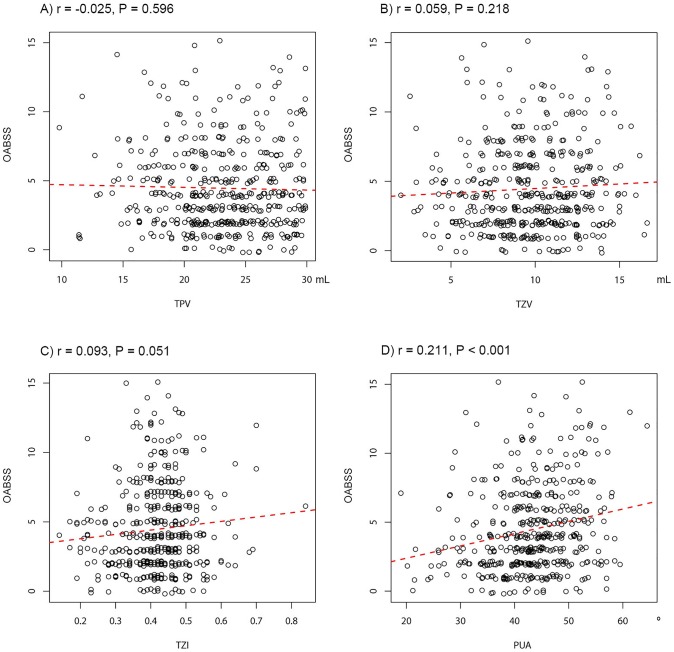
Correalation between prostatic anatomical factors and the Overactive Bladder Symptom Score (OABSS). (A) total prostate volume (TPV), (B) transitional zone volume (TZV), (C) transitional zone index (TZI), and (D) prostatic urethral angulation (PUA).

**Figure 5:**
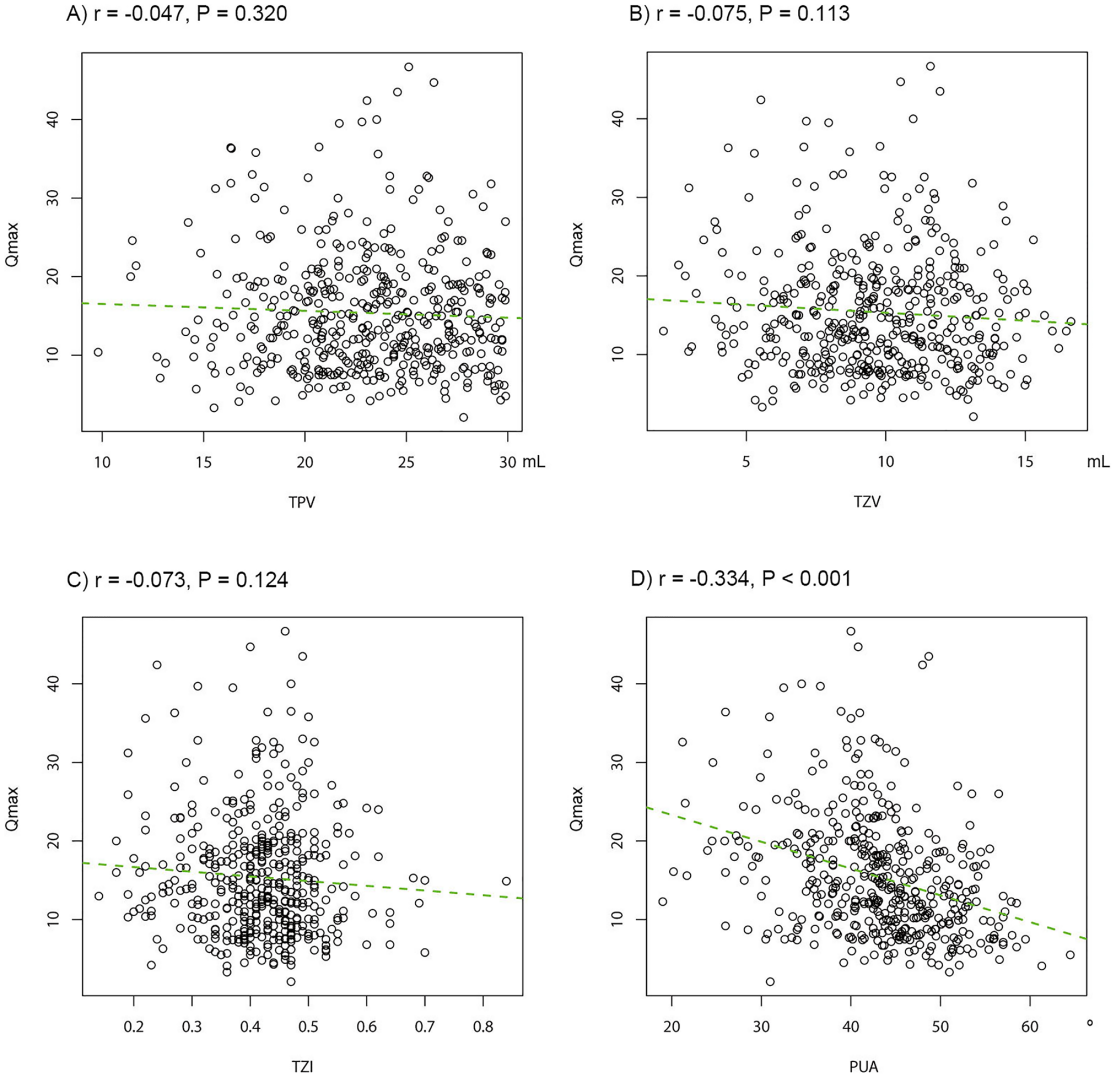
Correalation between prostatic anatomical factors and the peak flow rate (Qmax). (A) total prostate volume (TPV), (B) transitional zone volume (TZV), (C) transitional zone index (TZI), and (D) prostatic urethral angulation (PUA).

In Pearson's product-moment correlation, PUA (r = 0.269, P<0.001), TZV (r = 0.160, P<0.001), and TZI (r = 0.109, P = 0.022) positively correlated with IPSS (Fig. 3). In the subgroup analysis of the IPSS, PUA, TZV, and TZI also significantly associated with the voiding symptom domain and storage symptom domain. PUA and TZI significantly associated with the post-micturition symptom domain (Table 2). Only PUA correlated with the OABSS (r = 0.211, P<0.001) and the Qmax (r = −0.334, P<0.001); PUA correlated positively with the OABSS and negatively with Qmax (Fig. 4 and 5).

**Table 2:** Correlation coefficient between voiding parameters and prostatic factors in Pearson's correlation analysis.

	IPSS				OABSS	Qmax
	Total	Post-micturition symptom	Voiding symptoms	Storage symptoms		
Age	0.163**	−0.015	0.108*	0.235**	0.264**	−0.334**
TPV	−0.011	−0.088	0.018	−0.005	−0.025	−0.047
TZV	0.109*	0.032	0.107*	0.096*	0.059	−0.075
TZI	0.160**	0.115*	0.139**	0.132**	0.093	−0.073
IPP	0.044	0.058	0.068	−0.010	0.010	−0.086
PUA	0.269**	0.218**	0.250**	0.191**	0.211**	−0.334**

*, P<0.05;

** P<0.01.

Abbreviated as in Table 1.

### 4. Multivariate linear regression analysis

In a multivariate linear regression analysis, PUA (P<0.001) and age (P = 0.015) were independently associated with IPSS. In the subgroup analysis of the IPSS, only PUA significantly associated with the voiding symptom and post-micturition symptom domain, and PUA and age significantly associated with the storage symptom domain (Table 3). PUA and age correlated with the OABSS and Qmax independently (PUA: P<0.001 for OABSS and Qmax; age: P<0.001 for OABSS and Qmax) (Tables 4 and 5). The prostatic anatomical factors TPV, TZV, TZI, and IPP did not associate with IPSS, OABSS, or Qmax.

**Table 3:** Relationship between IPSS and prostatic factors in multivariate linear regression analysis.

IPSS total			
Factor	Standardized Coefficient β	t value	P value
Age	0.075	2.439	0.015
TPV	−0.075	−0.212	0.832
TZV	−0.146	−0.170	0.866
TZI	14.256	0.740	0.460
IPP	0.481	0.185	0.853
PUA	0.252	5.611	<0.001

Post-micturition symptom			
Factor	Standardized Coefficient β	t value	P value
Age	−0.001	−1.290	0.198
TPV	−0.036	−0.465	0.643
TZV	−0.050	−0.268	0.789
TZI	3.250	0.770	0.442
IPP	0.054	0.953	0.341
PUA	0.050	5.078	<0.001

Voiding symptoms			
Factor	Standardized Coefficient β	t value	P value
Age	0.020	1.259	0.209
TPV	0.096	0.498	0.618
TZV	−0.340	−0.729	0.466
TZI	12.533	1.200	0.231
IPP	0.095	0.674	0.501
PUA	0.125	5.149	<0.001

Storage symptoms			
Factor	Standardized Coefficient β	t value	P value
Age	0.063	4.359	<0.001
TPV	−0.135	−0.814	0.416
TZV	0.244	0.608	0.543
TZI	−1.529	−0.170	0.865
IPP	−0.101	−0.831	0.406
PUA	0.077	3.654	<0.001

Abbreviated as in Table 1.

**Table 4:** Relationship between OABSS and prostatic factors in multivariate linear regression analysis.

OABSS			
Factor	Standardized Coefficient β	t value	P value
Age	0.069	5.141	<0.001
TPV	−0.221	−1.438	0.151
TZV	0.420	1.126	0.261
TZI	−7.137	−0.853	0.394
IPP	−0.268	−0.238	0.812
PUA	0.079	4.068	<0.001

Abbreviated as in Table 1.

**Table 5:** Relationship between Qmax and prostatic factors in multivariate linear regression analysis.

Qmax			
Factor	Standardized Coefficient β	t value	P value
Age	−0.157	−5.026	<0.001
TPV	−0.278	−0.769	0.442
TZV	0.780	0.893	0.372
TZI	−19.311	−0.985	0.325
IPP	−2.722	−1.030	0.303
PUA	−0.309	−6.768	<0.001

Abbreviated as in Table 1.

### 5. ROC curves and cut-off value of PUA

**Figure 6:**
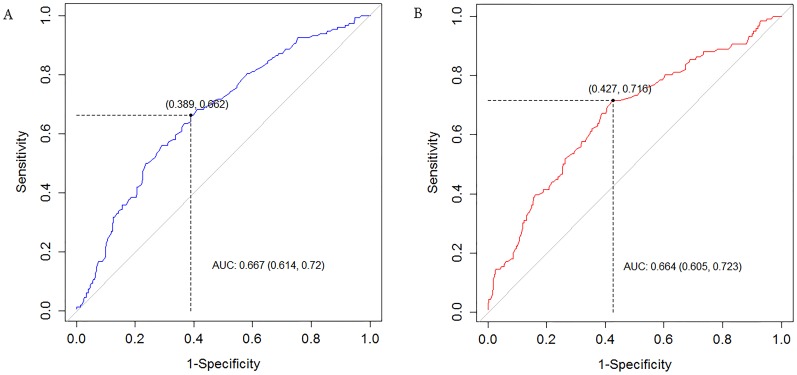
Receiver Operator Characteristic (ROC) curve of prostatic urethral angulation (PUA). (A) For IPSS 20 or above, Area under the ROC curve of PUA = 0.667, cut-off value = 43.7° (B) For Qmax 10 mL/s or less, AUC of PUA = 0.664, cut-off value = 43.5°.

For severe LUTS presenting with an IPSS 20 or greater [13], the area under the ROC curve (AUC) of the PUA was 0.667 and the cut-off value was 43.7°. In contrast, in cases where Qmax was 10 mL/s or less, the AUC of the PUA was 0.664 and the cut-off value was 43.5° (Fig. 6).

## Discussion

Although several researchers have emphasized the positive relationship between male LUTS severity and other prostatic anatomical factors such as IPP and TZI, there is no available data on such relationship among patients with LUTS and small prostate volume. In the clinical settings, quite a part of men with LUTS have small prostate volume. In our database, the proportion of patients with TPV 30 or less reaches approximately half. In this group of patients, there has been no remarkable anatomical prostatic factor related with urinary symptom and Qmax. As expected, TPV, TZI, TZV and IPP are not associated with urinary symptom and Qmax in multivariate analysis. Herein, we demonstrated that PUA is the only significant anatomical prostatic factor associated with subjective urinary symptoms as well as the uroflowmetric values.

Research into the significance of the PUA has recently increased. We first suggested in 2008 that the PUA is a causal factor of LUTS/BPH based on a fluid dynamic model and mathematical simulation. Subsequently, we also showed that the PUA significantly associates with the Qmax and symptom scores in men with LUTS [8], [14], [15]. In a retrospective study of 270 LUTS/BPH patients by Park *et al.* [16], an increased PUA is associated with an aggravation of voiding symptom. Ku *et al.* [7] suggested that the PUA correlated with bladder outlet obstruction in those patients with LUTS/BPH. In contrast to our results, however, no significant relation between PUA and LUTS severity or Qmax was shown. This contradictory result can be explained by the difference in study population. Only first-visit patients complaining of general LUTS were included in our study, while the study cohort in Ku's study was the men underwent a pressure-flow analysis.

In the current study, we demonstrate that the PUA is closely connected with LUTS severity and reduced Qmax by showing results similar to a previous study [8]. However, the current study is more compelling than previous studies due to several differences. First, the analysis was limited to patients with a small PV, specifically 30 mL or less, to isolate the effect of PUA on LUTS severity and Qmax with minimal impact from the PV. Second, ROC curves and cut-off values of PUA for severe IPSS and low Qmax are provided. These results are the first ever reported and are unique findings for the evaluation of LUTS/BPH. Meanwhile, the AUC and cut-off value of PUA as a significant factor of severe IPSS and for low Qmax are very similar (AUC: 0.667 vs. 0.664, cut-off value: 43.7° vs. 43.5°). Accordingly, a PUA of 43.5° or greater may be associated with both severe LUTS and low Qmax patients with small PV. Considering that low Qmax (10 mL/s or less) suggests urodynamical obstruction, a higher PUA (more than 43.5°) may indicate a significant obstruction of the prostatic urethra despite the relatively small PV.

The proportion of LUTS-presenting patients with a small PV was larger than physicians expected; in our institution, 51.8% of patients (444/857) presenting with LUTS had a small PV (Fig. 1). Many physicians have been interested in the cause of LUTS in patients with small PV, because a small PV suggests BPO is unlikely. Generally, other factors such as bladder problems, including detrusor hypoactivity, hyperactivity, hypersensitivity, and other problems, are emphasized. The potential role of the prostate has been overlooked in the pathophysiology of LUTS with small PV. Nonetheless, a higher bladder neck in men without lateral or median lobe enlargement can be seen by cysto-urethroscopy; many urologists believe that a higher bladder neck might be a causal factor of bladder outlet obstruction and LUTS. Increased PUA on TRUS corresponds to a high bladder neck on cysto-urethroscopy. While the pathophysiologic links between PUA and urinary symptoms/Qmax still remain uncertain, several explanations are possible. Previously, the PUA was proposed as a theoretical factor in LUTS; because the prostatic urethra is a bent tube, energy loss proportional to the PUA could occur during micturition and decrease urine velocity [14], [15].

Recently, Rodriguez-Nieves and Macoska introduced the remarkable theory that pathobiology other than androgen-mediated proliferation and smooth muscle dysfunction, such as inflammation and fibrosis, might contribute to the development and progression of LUTS [17]. According to this theory, urinary tract infection, prostatitis, aging, and type 2 diabetes mellitus could all cause tissue inflammation that promotes fibrosis in the lower urinary tract, and periurethral tissue fibrosis also can cause male LUTS. The role of the PUA must be addressed in conjunction with periurethral fibrosis. In young men with a healthy and compliant prostatic urethra, the PUA's role in the development of LUTS is limited, because such a prostatic urethra and bladder neck can adjust into a funnel-shaped inlet to ensure urinary flow during micturition. However, such an adjustment of the bladder neck and prostatic urethra is difficult to achieve by the fibrotic and non-compliant prostatic urethra of an elderly man. Presumably, a greater PUA might play a more pronounced role in the development of LUTS, especially in patients with periurethral fibrosis that occurs as a consequence of aging, infection, and metabolic syndrome.

According to the theory that BPH occurs in the transition zone or in the periurethral region [18], research into the TZV and TZI has been performed [19]. TZI (TZV/TPV) is a parameter that correlates significantly with evaluated parameters of BPH and may serve as a useful proxy for evaluating worsening obstruction. Several studies report that the TZI correlates not only with subjective voiding symptoms of patients but also with objective parameters such as Qmax [20]–[22]. In our study, IPSS correlated poorly with TZV and TZI, and there was no relationship in the multivariate regression test. This lack of correlation may result from the limiting of our cohort to patients with small PV. IPP is the vertical distance from the protruded tip to the circumference of the bladder at the prostate base taken at the mid-sagittal view. Chia *et al.* [11] presented IPP as a good predictor of BPO, even though IPP did not predict LUTS severity or reduced Qmax to a significant degree in our study, perhaps because the mean IPP value of our study cohort was very low and more than half the cohort were men with no IPP (0 mm); the IPP of almost all the patients (n = 441, 99.3%) was grade I due to small PV. Although IPP and TZI may be informative values in the evaluation and management of men with LUTS in general, such measurements are not useful in patients with small PV.

Our study has some limitations. First, the prostatic urethral anatomy may be altered during voiding; because the PUA measurements are performed while the patient is at rest, these measurements cannot reflect the PUA on voiding. Second, we could not exclude LUTS caused by bladder dysfunction or neurologic disease because no urodynamic study was performed; best efforts were made to exclude neurogenic bladder dysfunction by gathering a detailed patient history. But the sample of men is still subject to bias as all men had presented with LUTS. It is also regretful that a pressure flow study to evaluate obstruction was not performed. Although we suggested the relationship between PUA and urinary symptom and Qmax, the clinical significance of PUA might be limited at present. However, this can provide background of future researches on PUA, and ultimately the impact of PUA on response to medical treatment and disease progression should be tested, and it can suggest more important clinical significance.

## Conclusions

Of the prostatic anatomical factors analyzed, only PUA was significantly associated with urinary symptom severity and Qmax in men with LUTS and small PV. The PUA should be considered an important clinical factor in the evaluation and treatment of male LUTS. These observations should be investigated further through a larger scale prospective study. Furthermore, the impact of PUA on response to medical treatment and disease progression needs to be investigated. our results can provide a theoretical basis for transurethral resection or incisions of the prostate (bladder neck) in patients with small PV if unresponsive to the medical management.
